# Experiencing good results promotes positive feelings to high-intensity exercise among young adults: A qualitative study

**DOI:** 10.3389/fspor.2022.959079

**Published:** 2022-11-16

**Authors:** Kjetil L. Høydal, Eli-Karin Sjåstad Åsebø, Silje Louise Dahl

**Affiliations:** ^1^Department of Physical Education, Faculty of Arts and Physical Education, Volda University College, Volda, Norway; ^2^Department of Social Work, Faculty of Social Science and History, Volda University College, Volda, Norway

**Keywords:** endurance training, exercise intensity, physical activity, motivation, physical fitness

## Abstract

**Introduction:**

From a public health perspective, it is important to gain more insight into how people can be motivated to maintain effective exercise routines. It is a common belief that moderate-intensity exercise is more pleasant and enjoyable than high-intensity training. This study aims to provide insight into (1) participants' expectations and preferences for training intensity prior to training, (2) how longer-term participation affect participants' experience of endurance training with continuous moderate-intensity training and high-intensity interval training.

**Materials and methods:**

A total of 22 participants (14 women and eight men) between the ages of 21–30 volunteered for participation. Participants were randomized and divided into two equal groups. A total of 17 participants, nine women and eight men, completed the study. One group did moderate-intensity longer-lasting training and the other did high-intensity interval training. All participants completed three training sessions per week for 8 weeks. Semi-structured interviews were conducted with each participant before and after completing the training intervention. Data was analyzed using thematic analysis. This study is a part of a larger study evaluating and comparing the effects on endurance capacity of high-intensity interval training and moderate-intensity training. Physiological data are previously published.

**Results:**

The results describe participants expectations prior to training, and how they experienced the actual training. The overall experience of training comprises several factors that work together. Both expectations and actual experiences (e.g., of physical pleasantness or unpleasantness, of positive or negative emotions, and of actual results from the training) contribute to the participants' overall experience of exercise.

**Conclusion:**

The major finding is that improved physical fitness was a stronger motivator than feelings of pleasantness. Experiencing good results seemed to downplay feelings of unpleasantness and reinforce positive feelings toward exercise. Lack of results reinforce negative feelings toward exercise. Participants reported high-intensity exercise as more unpleasant and exhaustive, but the interval training group were more satisfied and experienced the training as more motivating.

## Introduction

Regular physical activity improves physical fitness and promotes good health (1). Health benefits are achieved both through low-to-moderate- and high-intensity training (1–4). Still, a growing body of knowledge suggests that high-intensity interval training provides larger physiological adaptations in both active recreational adults (2, 5–7) and patients (2, 8). High-intensity interval training stimulate larger physiological adaptations despite considerably less time spent and a lower exercise volume (9). From a public health perspective, these findings are important because lack of time is a frequently reported barrier to physical activity (10, 11).

Given the superior physiological effects of high-intensity training (2, 5, 8, 9), this training should be overall recommended. However, physiological adaptations to exercise rapidly decline when the activity level is not maintained or when participation ceases (12).

Interval protocols vary in terms of intensity, length of work periods, number of intervals and the length of rest periods (13, 14). Different protocols may be experienced differently, and this complicates the debate on whether high-intensity interval training is a viable public health strategy. The amount and variation of different protocols covered by the term “high-intensity training” makes it difficult to establish a clear answer to how high-intensity training is experienced (13, 15).

When advising recreationally active adults, experiences of pleasure or displeasure should be taken into consideration. Pleasure and displeasure refer to the positive and negative valence of feelings, they are core processes that accompany emotion, motivation, and bodily states. Pleasure involves feelings of enjoyment, happiness, and satisfaction, while displeasure in contrast involves dissatisfaction, disgrace or disfavor (16). Here, it is important to note that common belief is that moderate-intensity exercise is more pleasant and enjoyable (17), and that intensity below the anaerobic threshold is more pleasant than exercise above the threshold (17–20). This shift toward more negative feelings when intensity increases above the lactate threshold has been explained through the dual mode theory (21). According to this model, homeostasis is maintained when exercising with low intensity, and physiological adjustments largely occur outside awareness. When intensity increases, homeostasis is disrupted, and interoceptive information increase exponentially, as accumulating lactate stimulate free nerve endings, respiration becomes quicker and deeper, and additional non-oxidative muscle fibers are recruited disrupting coordination patterns (21). These negative feelings may deter future exercise participation (20, 22). However, others have reported higher degree of enjoyment after high intensity intervals (90% VO2max) compared to continuous moderate-intensity training (70% VO2max) (8, 13, 23, 24). A recent meta-analysis (25) reported affective response of high- intensity training to be experienced less positive during exercise compared to moderate- intensity training, whilst high intensity was experienced as more positive post-exercise. Together, the literature on experienced pleasure and displeasure at different levels of intensity is inconclusive.

There is a lack of longer-term intervention studies comparing pleasantness and enjoyment from moderate and high intensity training. Two reviews (13, 18) and a meta-analysis (25) on the field shows that studies have primarily measured the acute experience from single exercise sessions. One systematic review (22) included longer-term intervention studies, however only continuous protocols are examined. One study (26) reported that longer-term benefits, such as quality of life, motivated participants to future exercise. Their findings indicate that long-term effects of training may affect participants' motivation. However, differences in exercise intensity were not the subject of research. To the best of our knowledge, no other studies have examined this relationship thoroughly and interviewed participants before and after training interventions while comparing high-intensity training with moderate-intensity training. Therefore, an unanswered question is how longer-term participation affects participants' experiences of endurance training with high or moderate intensity.

This study aims to provide insight into (1) participants' expectations and preferences for training intensity prior to training, (2) how longer-term participation affect participants experience of endurance training with continuous moderate training and high-intensity interval training.

## Materials and methods

This study is a part of a larger study evaluating and comparing the effects on endurance capacity of high-intensity interval training and moderate-intensity training. The physiological effects of the project have been previously published (6). In the current sub-study, we used qualitative interviews to explore participants' expectations, experiences, perceptions, and motivations related to training with the two different intensities.

### Participants

Twenty-two participants (14 women and eight men, 21–30 years old) volunteered to participate through announcements in the local newspapers and on the university's web pages. In the first contact, principal investigator explained the study to the participants and emphasized the voluntarily and anonymous character. After inclusion, participants were randomized into two equal groups. Based on self-reporting, the inclusion criteria for the participants were (1) absence of any known disease; (2) no exercise limitations; (3) age between 18–30 years and (4) non-smoking. All participants took part in endurance training and other active leisure time pursuits at least once a week. They also had experience with running on a treadmill. A total of 17 participants, nine women and eight men, completed the study. Two participants in the interval training group (ITG) and three in the moderate continuous training group (MCTG) dropped out of the study due to illness, injury or personal reasons not related to the study. The participants have been given fictitious names to protect their anonymity during this investigation. See Table 1 for an overview of the 17 participants included in the analysis. Informative richness and data saturation was achieved.

**Table 1:** Shows participants pseudonyms, gender, training group, preferred training intensity and frequency of training prior to intervention.

**Pseudonyms**	**Gender**	**Training program**	**Preferred training intensity**	**Frequency of training (sessions per week)**
Joe	Male	Continuous run	No preference, like both	1–2
Nils	Male	Continuous run	Moderate	2–3
Elisa	Female	Continuous run	High	1
Nina	Female	Continuous run	High	1–2
Ane	Female	Continuous run	Moderate	1–3
Siri	Female	Interval	No preference, like both	1
Cecilie	Female	Continuous run	Moderate	1
George	Male	Interval	High	3
Peter	Male	Interval	No preference, like both	2–3
Ina	Female	Interval	High	3
Martha	Female	Continuous run	No preference, like both	3
Simon	Male	Interval	High	2
Kevin	Male	Interval	Moderate	2–3
Henry	Male	Continuous run	No preference, like both	1
Anne	Female	Interval	High	2
David	Male	Interval	Moderate	1
Emilie	Female	Interval	No preference, like both	1–2

The Regional Committee for Medical Research Ethics approved this study (Ref. 2010/2959), and it conforms to the declaration of Helsinki (27). All participants reviewed and signed informed written consent prior to participation and had the opportunity to withdraw from the study at any time.

### Training protocols

The project continued for 10 weeks from pre-test to post-test including a total of 8 weeks' training. Both groups performed three training sessions per week. All training sessions were performed on a treadmill. Participants were allowed to listen to music and there was no restriction regarding social interaction between participants, within the same training group, exercising together. The two training groups (MCTG and ITG) were separated to avoid being influenced by each other. To ensure that the intensity of exercise was appropriate, we measured the heart rate using Polar Accurex heart rate monitors (Polar Electro, Finland) in each session. Participants controlled the speed of the treadmill themselves, based on the heart rate monitor. When the heart rate started to increase (drift) in the MCTG, the speed of the treadmill was reduced to secure the target intensity. All training sessions were performed in our laboratory and were supervised by an exercise physiologist, who made sure that both training groups trained in their prescribed intensity zones.

In both training protocols the exercise started with a 10-min warm-up and finished with a 3-min cool-down at 70% HR_max._

- MCTG: continuous running at 75% of HR_max_ for 62 min. A total of 3 h and 45 min per week exercising.
- ITG: 4 x 4 min interval training at 90 to 95% HR_max_ with 3 min active resting periods at 70% HR_max_ between each interval. A total of 1 h and 54 min per week exercising.

### Interviews and analysis

Semi-structured interviews (28) with each participant were conducted before and after completing the training programme. Pre-intervention interviews were conducted prior to randomization. Semi-structured interview guides were developed for pre- and post-training to allow the participants to tell their stories openly in their own words (28). The interview guides were developed by the authors for the purpose of the study through a review of the literature on endurance training and motivation. The pre-intervention interview guide focused on the following main topics: (1) Training background (2) Physical fitness, (3) Training habits, (4) Motivation for exercise, (5) Knowledge about exercise, (6) Expectations for the training intervention. The post-training interview guide focused on the following main topics: (1) Subjective experiences of the training, (2) Physical fitness, (3) Knowledge about exercise, (4) Motivation for exercise, (5) Future training goals (Supplementary material). Prior to the data collection, pilot interviews were conducted, and smaller adjustments were made.

The second author (Assistant Professor in sport psychology and pedagogy) conducted all 34 interviews in a neutral environment. Field notes were taken by the same author during interviews. Interviews were audio-recorded and transcribed verbatim. Findings were presented for the participants, and all participants were given the opportunity to provide feedback. Interviews prior to the intervention lasted 25–70 min (Mean = 43 min) and post-intervention 22–42 min (Mean = 31 min). During the last interviews, data saturation seemed to have been reached, as no new information nuances were identified (29). Quotations used in the text are translated to English language.

We approached the data from a critical realist perspective, where language is understood as constructing our social realities, but confined by possibilities and constrains in the material world. Critical realism takes an intermediate position between positivism and constructivism, as an epistemological approach that unites quantitative and qualitative logic (30).

Initially, transcripts from the interviews before and the ones after the intervention were analyzed independently. Subsequently, the pre- and post-test transcripts were compared. Interviews were analyzed using thematic analysis (31). This mode of analysis is flexible, has potential to provide rich and complex understandings, and serves well to obtain rigor in accordance with Morse (32). Two researchers (First author, male associate professor in exercise physiology, and second author, female assistant professor in sport psychology and pedagogy) worked through the transcripts, applying emergent codes that characterized significant statements. This helped us to familiarize with the material and made it easier to retrieve important sections later. In the next step, we grouped the initial codes in themes relevant to the research questions of this article: expectations prior to training, experiences of training with high intensity and with moderate intensity, and motivation. Finally, detailed analyses of the themes were conducted, and we selected suitable quotes. Writing up the results section, we reviewed and revised themes, codes and text to ensure coherence between the material and the text.

Themes and interpretations were discussed between all researchers; First author, second author, and third author, (female associate professor in sociology, with expertise in qualitative methods) to obtain different perspectives and opinions on the data. Through discussions within the research team, consensus was reached upon codes and themes. We used MAXQDA (Pro VERBI Software, 2016) in the analytic process.

## Results

Prior to training three main teams were generated: (Un)pleasantness, good results and not knowing how. Post training, we generated two main teams: (Un)pleasantness and good results.

Our data suggest that the overall experience of training comprises several factors that work together, and that both expectations (e.g., of physical pleasantness or unpleasantness, of emotions, and of results from the training) and actual experiences (e.g., of physical pleasantness or unpleasantness, of positive or negative emotions, and of actual results from the training) contribute to the participants' overall experience. Although the themes are presented separately for analytic purposes, we acknowledge that they are interwoven and work together to form the whole.

### Participants' expectations prior to training period

The participant's expectations of preferred exercise intensity prior to the training period did not point in a particular direction. Six participants preferred to take part in high-intensity training, five preferred to take part in continuous moderate-intensity training, and six of the participants had no preferences in terms of training intensity (Table 1).

### (Un)Pleasantness

Those who preferred moderate intensity, emphasized feelings of pleasantness during training sessions. Cecilie, for example, said that she preferred training sessions where she was able to “*talk a little”*. Nils preferred “*a comfortable pace*” and described moderate-intensity training as “*more motivating*”. Kevin also preferred moderate-intensity training, and he associated high-intensity training with a certain degree of pain:

*[The interval training] will probably be painful now and then… I know that the moderate-intensity training will not be very hard, but [doing] intervals… that will burn! I guess it tears the muscles more, perhaps*.

Moderate-intensity training was something Kevin was used to and knew he could handle. Interval training, on the contrary, was somewhat unfamiliar and he expected it would be more straining on the body.

What the participants perceived as pleasant or unpleasant, and how much they weighted these feelings, varied. Some found the feeling of exhaustion highly uncomfortable and tried to avoid it. Others emphasized how training to exhaustion gave them a good feeling after the session and described this as more important than how they felt during training. Nina and George described the latter preference:

*It might not be pleasant while you are doing it - you might not be so happy then, but when you are finished, it is the best, I think!*

*To come home and sit on the couch …and feel that you are still tired and breathe a little heavy… than I know it has been a good exercise*.

To some, the feeling of exhaustion was even more important—and positively valued. George, for instance, described the feeling of being exhausted as a main reason for why he preferred high-intensity training. Elisa's described a good training session as one where she “gave it all”:

*It is when I feel that I get really tired, when I feel that I take it all out and sweat in a way. In the end I feel that I could not give more*.

To the participants who stressed the feeling following exhaustion as pleasant, feelings of discomfort during exercise was not emphasized and rather described as subordinate.

### Good results

All participants reported experiencing good results, i.e., improved physical fitness, as an important factor for their motivation to exercise. For instance, Siri explained it this way when asked about her expectations:

*I like intense training better. I guess I feel that results come faster with this kind of training. What I hope for, is that the good feeling related to exercise will return and that I'll feel that I can manage more gradually*.

Siri had experienced good results from high-intensity training earlier and that the results she had experienced before was an important motivating factor for this kind of training regime. Others had no clear preference for high- or moderate-intensity training, but they emphasized the importance of experiencing positive physical effects:

*When it comes to high- or moderate-intensity, I can't really see a large difference. I think what motivates me is that I want to see results*. (Henry)

*The goal is to be in better shape at the end of the period than I am before the training starts. … I feel that my shape never improves a lot, and I exercise all the time*. (Marta)

Improved physical fitness was something that many of the participants valued and expected to achieve. Many of them did however report that they had little knowledge and were insecure of how to achieve their goal.

### Not knowing how

Several participants reported lack of knowledge related to exercise. They were uncertain of how to train effectively and what they could expect from exercise in terms of improved physical fitness. In addition, they were uncertain of how to train safely and how hard they ought to push themselves. Anne explained her lack of knowledge and what she hoped to achieve by participating in the study:

[My knowledge of exercise] …* Is not very high… I don't know which exercise that is effective. […] I hope to increase my knowledge about training methods and my own shape, and limitations and possibilities [for endurance training]… and how hard I can push myself*.

The participants' uncertainty was particularly pronounced for high-intensity training, and some said that they feared that interval training might overstrain their bodies and lead to injuries. Several of the participants considered moderate intensity a safer choice, and this influenced which intensity they preferred when exercising on their own. David explained his preference for moderate-intensity training like this:

*[I prefer] moderate intensity, but it may be because I don‘t know how to exercise. I have never had a conscious relation to how I should exercise… If high intensity is the best? […] If I am to exercise at high-intensity, I feel that I should know what I'm doing, sort of*.

The notion that high intensity training required more knowledge than other endurance training regimes, and that this was knowledge he lacked, made him stick to what he considered the safer choice.

### Participants' experiences post exercise period

#### (Un)pleasantness

In the same way as participants' expectations prior to training were ambiguous, so were their actual experiences of the training in retrospect. Most participants performing the moderate intensity with a long duration described the training as light, and no one characterized it as physically unpleasant or exhaustive. However, many mentioned boredom and described the training as demotivating. Feelings of boredom seem to be closely related to time spent, i.e., long duration, and the sensation of not exercising properly. Participants in the moderate training group reported that they did not feel exhausted during exercise and felt they could have managed a higher load. Both factors were frequently mentioned as having a negative impact on their emotions related to the exercise. Although not *physically* unpleasant, the training was experienced as *mentally* challenging and unpleasant.

The most common reflection among the participants in the moderate training regime was that the training was okay initially, but that there was a shift during and toward the end of the training period. Elisa's description was typical:

*It was quite all right. Really okay. That is, at the start, it was really okay. And then, the last weeks, it became somewhat boring and tedious*.

Cecilie described an even more profound change in her motivation:

*In the beginning, I thought it was motivating, funny and exciting. Gradually it became heavier… Gradually I lost the motivation… It became mentally heavy to attend the training… even though it was not physically exhausting or anything*.

In the ITG, on the other hand, many described the training as very hard and exhaustive, but also “*exciting*” and “*fun*”. For instance, George described the training as varied but “*hard all the time*”. Simon elaborated: “*the sessions are unpleasant. It is hard training… You are about as tired as you can be, almost… But, really it has gone well*”. Kevin summed up his experience in a positive note, describing the interval training as “*very much fun, really*”.

There was a clear shift in the participants' experiences of the training during the intervention period. This shift was particularly pronounced in the moderate training group. The most typical pattern described was that the feeling of boredom, and thus negative emotions, developed gradually during the training period. Elisa expressed it as follows:

*In the beginning, it was okay, but toward the end of the project [,] it became somewhat boring and tedious. Maybe because I walked on a moderate heart rate and didn't feel that exhausted, and at the same time it lasted for a long time*.

In her case, it seemed that the moderate level of strain made her feel that she did not exercise “*properly*”. This reinforced the notion that the training sessions were of long duration and increased her sense of boredom. Joe described “*mixed feelings*” toward the moderate-intensity regime. Although he appreciated the feeling of coping, he described that not pushing himself made the training boring:

*At times boring, really, but at the same time, it was two-fold. It was lovely to step off the treadmill after 75 minutes. Mentally, I felt that was good. [...] I felt I could have managed a higher load. You don't feel that you are pushing your body, so that's why the second half gets quite boring. I don't know. I have mixed feelings about that training*.

Others described how they “*counted the minutes*” and felt that they had to jog for “*an eternity*”. Frequently, the moderate-intensity regime was characterized as being a “*mental challenge*” demanding a high-degree of “*willpower*” “*because it lasted so long*”.

In contrast, participants in the ITG stated that the short duration was motivating. The short time spent made the hard training sessions easily manageable:

*Because the session only lasted half an hour, I knew I could manage, no matter how exhausted I was*. (Peter)

*It was motivating and quickly done. It was a very effective training method*. (Anne)

To sum up, both the feeling of (not) training properly and the duration of the training sessions influenced the participants' experiences of pleasantness or unpleasantness related to training. The shift toward negative emotions was more pronounced among those exercising at moderate-intensity longer-lasting training.

### Good results

Prior to training, all participants expressed that expectations of improved physical fitness were a major motivating factor for the training. The post-intervention interviews largely confirmed the importance of outcome: The experienced effect of the training affected their feelings related to the training. When participants experienced good results, negative feelings, such as the training being hard and unpleasant, were downplayed and tolerated well. When the effect of the training was modest or flattened out, the negative emotions were emphasized more. However, if they again experienced good effect, their feelings associated with the training also became more positive.

For instance, Siri expected the high-intensity training to provide fast results, and therefore, she preferred this regime. After the training period, she described the training as exciting, fun and exhaustive. During a period when the effect of training flattened out, i.e., she was not able to increase the speed during the intervals, and the positive results were less profound than she had expected, she had been disappointed:

*I had a good start, and then it leveled off or fell a little, so I noticed that the motivation fell somewhat when the result was not as good as expected*.

George described a similar experience, finding it hard to “*motivate [him]self* ” during a period with modest progress. Even though both Siri and George described the training as hard, they expressed positive emotions toward it as long as the expected results occurred. When the results did not match their expectations, this negatively impacted their motivation. Still, when achieving progress again toward the end of the intervention period, their feelings became more positive again.

Others did not experience any drop in progress, and thus no negative change in the experience of exercise. Prior to the intervention, Kevin was skeptical toward high-intensity training because he expected it to be somewhat painful. After the training period, he was surprised that he had found the training “really fun actually”. He further explained:

*[I] had good progression in the start; probably because I had so bad stamina, it increased really fast […] It was heavy, really heavy. I'm the type that pushes myself to the max. In this case, I knew that if I pushed myself, I'd achieve good results and that was part of my goal with doing this thing*.

To Kevin, the good results he experienced were highly motivating, and it compensated for the unpleasantness he experienced during the sessions. Peter was positive to both high- and moderate-intensity training prior to the intervention. He expected the training to be hard and looked forward to “*push [him] self* ”. He explained his experience as follows:

*I experienced progress after each session, so that was positive… that I sort of managed to motivate myself to the training because I knew it worked*.

The feeling of positive results sparked Peter's motivation and made the strain bearable and worthwhile.

Participants in the MCTG did not experience the same increase in physical fitness, measured as maximal oxygen uptake and 3,000 meter performance (6), as the ITG, and this seemed to influence how they experienced the training. Although they did not find the training hard or physically challenging, the lack of expected results seemed to trigger negative emotions toward the training. This experience was described by several participants in the MCTG, and were summed up by Martha:

*I really struggled with the motivation. I think I'm a little restless by nature, and I'm quite competitive, so it goes against my nature to just keep going [at] the same speed. And then there was extremely slow progress in speed. I had hoped that in the end, I'd be able to run, but… It was demotivating to see that there was very little improvement*.

The lack of progress seemed to gradually take the joy out of the training for the MCTG-participants. Although many thought the training was ok at first, not getting the results they wanted altered their view of the training in a negative direction. Nils' quote was quite typical for the MCTG-group.

*It was ok the first four weeks, and then… well then, I didn't experience any progress, really. And, yes, during the two final weeks, I was tired of it*.

## Discussion

This study aims to provide insight into (1) participants' expectations and preferences for training intensity prior to training, (2) how longer-term participation affect participants' experiences of endurance training with continuous moderate training and high intensity interval training.

We found no clear patterns regarding the participants' preferences for exercise intensity prior to training (Table 1). Participants emphasized factors such as the training being time-efficient, expected results from the exercise, pleasantness and displeasure, fear of not being able to sustain the training over time and lack of knowledge. This is in accordance with factors previously reported as facilitators and barriers to participation in physical activity (15). Before the training, some participants preferred moderate-intensity training, because they anticipated the high-intensity training to be more unpleasant and even somewhat painful. Previous studies have reported greater pleasure from moderate-intensity training (17–19, 33), and we thus expected the majority to prefer this intensity. A considerable group did however, express that they enjoyed and preferred intense training, in accordance with (13). Some participants had no preference at all. Thus, participants' expectations were ambiguous.

Each participant gave several reasons for preferring either moderate or high intensity regimes. Still, the desire for achieving results from the training was the single reason emphasized by all participants, regardless of whether they preferred moderate- or high-intensity training or had no particular preference. Expecting gains in physical fitness was therefore important (34). Additionally, lack of knowledge about how to perform endurance training and the to-be-expected results of different regimes seemed to predict a preference for moderate-intensity training prior to the intervention.

Following the exercise prescriptions of moderate exercise does not necessarily provide similar good results in aerobic capacity as exercise with a higher intensity (1). Still, the American College of Sports Medicine (1) prescribe moderate intensity because it is expected to improve the adoption and adherence due to pleasantness. Our findings offer a different perspective on these recommendations. All participants in our study expressed that they expected marked results in physical shape and that this was an important motivating factor for training. Based on our data we propose that lack of knowledge and the gap between subjectively expected and experienced results, poses a threat to long-term adoption of endurance training. According to (35), self-regulation is the most important predictor of initial short-term adoption of physical activity. Knowledge of outcomes supports autonomy in the sense that individuals choose an activity based on their knowledge of the outcome and their personal valuation of certain outcomes from exercise. Recognizing that there are individual differences in response to training (36) is important. Still, clear recommendations of intensity may provide a better actual understanding of what results one can expect (2), and thus, reducing the gap between expectations and experienced results from training.

For long-term participation, intrinsic motivation, such as valuing the actual experience of exercise, is suggested to be more important (35). Our data conform to studies reporting training below the lactate threshold as more pleasant than training above it (17–19, 21). However, we also found that the ITG-participants' overall experience was positive, while many of the participants in the MCTG experienced a gradual fall in motivation throughout the training period. This reduced motivation seemed to be connected to both the sense of boredom, the long duration of the sessions and the modest results achieved from the training. Few studies have studied affect in interval exercise protocols (18, 22), and the prediction of the dual-mode theory (21) for these protocols is thus not clear (13). Our study does not measure affect *during* the exercise sessions, but rather the participants' experiences over the 2-month exercise period. Our data indicate that affective responses in interval protocols should indeed be investigated more thoroughly in future studies, in both single exercise sessions and longer-term interventions. Additionally, in line with (25) some participants reported that they appreciated the feeling they got after intense exercise, even if the feeling during exercise was somewhat unpleasant. Future studies should investigate feelings after exercise more thoroughly, and particularly in aerobic intervals protocols.

For the participants in the ITG, the short duration of the sessions was highlighted as a motivating factor. Shorter duration was preferred due to expectations of boredom and because high-intensity training was experienced as somewhat unpleasant. This contrasts the findings from (18) and (1) as we found that the short duration of training reduced participants' negative emotions when performing high-intensity training. The long duration of the moderate-intensity training sessions, on the other hand, contributed to participants' negative emotions toward this otherwise relatively comfortable training. Although the participants in the MCTG did not describe the training as physically unpleasant, the training was experienced as psychologically challenging and unpleasant.

The ITG improved their VO_2max_ by 11.3% as opposed to 4.7% for the MCTG (6). Our data indicate that the actual results of training moderate or reinforce the experiences of the exercise as either pleasant or unpleasant. When the participants experienced good results, they did not emphasize the negative feelings of unpleasantness. However, when they experienced less progression, they emphasized feelings of unpleasantness more strongly. The interviews with the participants largely confirmed that they found the exercise motivating because they experienced gain from it and not because it was inherently pleasant or enjoyable (34).

The absence of physical unpleasantness did not weigh up for the lack of results. This may indicate that identified self-regulation is also more important in long-term motivation for maintenance of exercise than previously reported (35). Our data shows that both expectations of results before training and experiencing results from training are important for motivation. Our findings are in such consistent with (26).

The major contribution of the present study is that it captures the changes in the experience of exercise over a longer period, rather than the acute affect in single exercise bouts. The qualitative interviews contribute with a richer understanding of people's experiences with both moderate and high intensity and the meaning they attach to their experiences.

Participants in our study are healthy young moderately trained. In line with (15) and (13), our data shows that high- intensity training can be well tolerated and even enjoyed. However, our findings cannot be generalized to inactive or other age groups. Previous studies on sedentary individuals have found that most individuals select and prefer to exercise at an intensity corresponding to lactate or ventilatory threshold (37, 38). However, the informal comments from the patients in (8, 24) indicate that high intensity interval protocols could be enjoyable even for sedentary and old participants. Further longitudinal studies are necessary to assess both affect and results of exercise together with motivation and adherence to training in various groups, including different age groups, various level of fitness and physical activity level. This further include designing exercise protocols with sufficient intensity-level to promote significant physiological effect of exercise, combined with the experience of pleasure during and post-exercise (39). Adding to this, other factors, such as training alone vs. training in groups, and how this affects the experience of intensity, and adherence, can be the focus of future studies.

## Strengths and weaknesses

The major strength of this study is that the interventional design offers the possibility to capture longer-term changes in experience and motivation than the acute experience of a single training session. For instance, our results show how experiencing progress from training influences the overall experience and motivation to train. However, limitations of the study include the environment in which the participants exercised. Participants reported the sterile environment in the training room as something they “*got tired of”*. This was evident for both groups and might have had a larger impact on the MCTG as they exercised with a longer duration than the ITG. Overall, generalization is a limitation for qualitative approaches, and care must be taken when applying our results to other groups. There is always a potential for bias in the inclusion process. In accordance with our inclusion criteria only healthy, non-smoking participants between the age of 18–30, with no exercise restrictions were recruited in this study. Participation in regular physical activity was not inclusion criteria, and no participants were excluded for this reason. Still, our sample consist of moderately active participants, exercising between 1–3 times per week (Table 1). It might be that participants who are motivated for exercise have signed up for participation. This could be a potential bias, which could make the data less transferable to the inactive part of the population. Nevertheless, previous studies on patients with metabolic syndrome and heart failure (8, 24) have reported similar findings.

## Conclusion

This study shows that expectations prior to training, the actual experience of training and the results participants perceive all contribute to their motivation to exercise. The major finding is that improved physically fitness is a stronger motivator than feelings of pleasantness. Experiencing good results seems to reduce feelings of unpleasantness and reinforce positive ones toward exercise. Lack of results, however, reinforces negative feelings toward exercise. Thus, although participants reported the high-intensity training as more unpleasant and exhaustive than the ones following moderate-intensity training, the ITG participants were more satisfied and experienced the training as more motivating.

## Data availability statement

The raw data supporting the conclusions of this article will be made available by the authors, without undue reservation.

## Ethics statement

The studies involving human participants were reviewed and approved by Regional Committee for Medical Research Ethics Central Norway, REK Central. The patients/participants provided their written informed consent to participate in this study.

## Author contributions

KLH: he was the in-charge of the entire research process, research idea, study design of the work, generating data through follow-up during intervention, analysis and interpretation of the data, main writer of the manuscript, and provided ethical approval for publication of the content. E-KSÅ: substantial contributions to the design of the work, generating data in interviews, analysis and interpretation of the qualitative data, drafting and revising the analysis critically for important intellectual content, and provided important contributions to the final manuscript. SLD: substantial contributions to the design of the work, analysis and interpretation of the data, drafting and revising the analysis critically for important intellectual content, and provided important contributions to the final manuscript. All authors contributed to the article and approved to the submitted version.

## Conflict of interest

The authors declare that the research was conducted in the absence of any commercial or financial relationships that could be construed as a potential conflict of interest.

## Publisher's note

All claims expressed in this article are solely those of the authors and do not necessarily represent those of their affiliated organizations, or those of the publisher, the editors and the reviewers. Any product that may be evaluated in this article, or claim that may be made by its manufacturer, is not guaranteed or endorsed by the publisher.
