# Supplementary material for: Experiencing good results promotes positive feelings to high-intensity exercise among young adults: A qualitative study
Source: Front Sports Act Living. 2022 Nov 16;4:959079. doi: 10.3389/fspor.2022.959079 (PMC9709123; doi:10.3389/fspor.2022.959079)
Supplement: Supplementary file 1 [file Data_Sheet_1.pdf]

Interview guide 1st interview (before the intervention)

- **Can you tell me a little about who you are?**
    - o Age
    - o Gender
    - o Marital status
    - o Education (degree)
    - o Profession
    - o Can you tell me something about how your life situation affects your training and your training opportunities?
- **Training background**
    - o Can you tell me something about your training background and physical activity level during the day?
    - o If so, what kind of sports or other activities? How long?
    - o Maybe why you are lucky? Why do you quit? Positive and negative experiences with training?
    - o Possibly in what way do you think this can affect this? Do you think it might have any influence? What motivated you then? How do you think it will be now?
- **Physical fitness and training:**
    - o Can you say something about how much you train? Intensity, duration, quantity and quality? Degree of physical activity? Level?
    - o Can you say something about how you train in general?
    - o Can you say something about how you train to increase your endurance?
    - o Can you say something about what intensity level you like to train at? Possibly why?
    - o Can you describe your view of your own physical shape?
    - o Do you see yourself as well trained? In what way? why not? What do you mean by that?
    - o Can you describe a good training week?
- **Knowledge of training:**
    - o How would you describe your own knowledge of training?
    - o Can you say something about how you acquire knowledge about training?
    - o How does knowledge about training affect your training? Do you have an example of this?
- **Training motive / motivation:**

o You said you trained… ..Why do you train? Can you say something about how you experience exercising?

o What factors are important for you to continue exercising? Time use, better shape, body vision, dieting, pleasurable activity or other factors?

o Are you motivated to exercise?

o Can you say something about what motivates you to exercise? Why? What do you mean by that? Can you elaborate on this?

o Do other people affect your motivation to exercise? Possibly how? Maybe why not?

o Have you seen your training goals? Interim goals? What do you want to achieve through training?

o Why is this important to you?

o Can you describe what motivational strategies you use to motivate yourself?

o What motivational strategies do you think you will benefit from during the training program and why?

o Can you say something about whether this exercise program will change your motivation to exercise?

• **Expectations related to the training intervention:**

o Why did you participate in this training intervention?

o Can you say something about what expectations you have for this training intervention?

o What do you want to achieve through this intervention?

o Previous experiences with this type of training?

o What, how and why?

Interview guide 2nd interview (after the intervention)

• **The training program:**

o Can you describe the training program you have completed?

• **Subjective experience of the training:**

o How did you experience the training? Hard, moderate, or easy? Pleasant or uncomfortable. Can you describe the feeling?

o Can you say something about what intensity you like to train on and why?

o How long do you like to train in this way?

- What form of exercise do you prefer?
- How do you experience the effect of the training?
- Possibly against previous training background: What do you think has the best effect?
- What is most motivating for you? In what way? Can you describe it? Can you elaborate on that?
- Can you say something about your benefits from being part of this training program?
- Can you tell me about your experiences related to the project? How have you experienced it? What are our possible challenges?
- What significance has this had for you? The value of training?

• **Physical fitness:**

o Can you describe your view of your own physical shape in relation to before you started with the exercise program?

o Degree of physical activity? Level? Intensity, duration, quantity and quality?

o Do you see yourself as well trained? how? In what way?

o What do you mean by that?

• **Knowledge Bout exercise:**

- How would you describe your own knowledge of training now?
- Can you say something about how did you acquire knowledge about training after you started with the training program?
- Can you say something about what you know about interval training with high intensity and continuous training with lower intensity and longer duration (long run)?
- What do you think the knowledge about training has for choosing a further training method?

• **Motivation for exercise:**

- Can you describe what motivational strategies you find useful during training and why? What has motivated you to keep going? How did you motivate yourself? Have you received help from others? Can you possibly describe how?
- Can you say something about whether this exercise program has changed your motivation to exercise?

o Can you say something about whether this is a form of exercise you want to continue with in the future? Possibly why / why not?

o What factors are important for you to continue exercising now?

o How do you see your own life situation now? Has the training affected your everyday life, or in what way?

o Has your life situation changed since you started the exercise program? Possibly how and why, why not? Have you observed changes in yourself through participating in this project? Can you possibly describe this change? Do you notice changes in everyday life?

o Can you possibly describe other positive ripple effects of the training?

• **Future training goals:**

o What kind of goals do you have now? Sub-goals, etc. Why, why not?

o How will you continue? Why do you want to do it this way?

o Would you recommend this type of training to others? why, why not?
